# The Role of Mitochondria in Optic Atrophy With Autosomal Inheritance

**DOI:** 10.3389/fnins.2021.784987

**Published:** 2021-11-15

**Authors:** Elin L. Strachan, Delphi Mac White-Begg, John Crean, Alison L. Reynolds, Breandán N. Kennedy, Niamh C. O’Sullivan

**Affiliations:** ^1^UCD Conway Institute, University College Dublin, Dublin, Ireland; ^2^UCD School of Biomolecular and Biomedical Science, University College Dublin, Dublin, Ireland; ^3^UCD School of Veterinary Medicine, University College Dublin, Dublin, Ireland; ^4^UCD Diabetes Complications Research Centre, Conway Institute of Biomolecular and Biomedical Science, University College Dublin, Dublin, Ireland

**Keywords:** mitochondria, retinal ganglion cells (RGC), optic atrophy, *in vivo* models, retinal organoids

## Abstract

Optic atrophy (OA) with autosomal inheritance is a form of optic neuropathy characterized by the progressive and irreversible loss of vision. In some cases, this is accompanied by additional, typically neurological, extra-ocular symptoms. Underlying the loss of vision is the specific degeneration of the retinal ganglion cells (RGCs) which form the optic nerve. Whilst autosomal OA is genetically heterogenous, all currently identified causative genes appear to be associated with mitochondrial organization and function. However, it is unclear why RGCs are particularly vulnerable to mitochondrial aberration. Despite the relatively high prevalence of this disorder, there are currently no approved treatments. Combined with the lack of knowledge concerning the mechanisms through which aberrant mitochondrial function leads to RGC death, there remains a clear need for further research to identify the underlying mechanisms and develop treatments for this condition. This review summarizes the genes known to be causative of autosomal OA and the mitochondrial dysfunction caused by pathogenic mutations. Furthermore, we discuss the suitability of available *in vivo* models for autosomal OA with regards to both treatment development and furthering the understanding of autosomal OA pathology.

## Introduction

Optic atrophy (OA) with autosomal inheritance is a heterogeneous neurodegenerative disorder primarily characterized by the bilateral degradation of axons in the optic nerve, leading to a progressive and irreversible loss of vision. OA is largely referred to in the literature as dominant optic atrophy, however, as we will discuss further below, both dominant and recessive forms of the disease are common. It is thought to be the most prevalent form of hereditary optic neuropathy, with an estimated prevalence between 1 in 25,000 to 1 in 12,000 in some regions (Toomes et al., 2001; Yu-Wai-Man et al., 2010a). Age of onset is most commonly during the first or second decade of life with diagnosis usually occurring during childhood (Lenaers et al., 2012; Ham et al., 2019). Autosomal OA has a complex symptomatology and the extent of vision loss is highly variable. Some patients experience moderate visual loss and color vision defects, while in others the visual loss is more severe resulting in blindness. Furthermore, approximately 25% of patients exhibit extra-ocular symptoms including ataxia, peripheral neuropathy, deafness, and myopathy which is referred to as syndromic-OA (Yu-Wai-Man et al., 2010b; Ham et al., 2019). As yet, there is not a clear understanding why some individuals develop syndromic-OA while others, in some instances with the same underlying genetic variations, develop non-syndromic-OA (involving visual symptoms only) (Ham et al., 2019). Although a small-scale, off-label trial has been conducted using the coenzyme-Q10 analog idebenone (Romagnoli et al., 2020), there is currently no treatment commercially available for autosomal OA, representing a significant unmet need for affected patients. Therefore, there is a clear need to further our understanding of the molecular events that give rise to this disorder so that we might develop additional treatment strategies to address autosomal OA which would have relevance for other neurodegenerative diseases. This review discusses the converging evidence for mitochondrial dysfunction underpinning axonopathy in autosomal OA. Furthermore, we examine current and potential model systems available for the study of autosomal OA, many of which would be highly amenable to adaptation for other forms of hereditary retinal and neurological disease.

**FIGURE 1:**
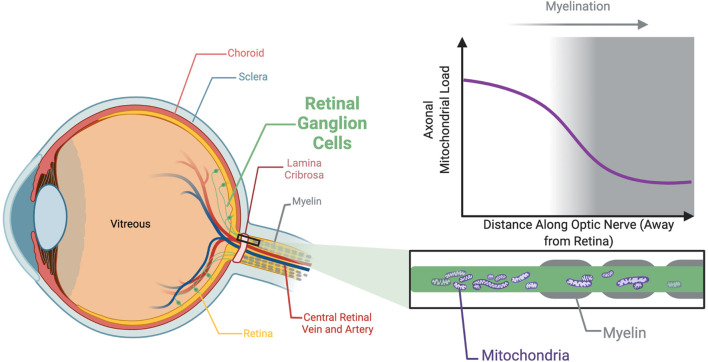
Unmyelinated axons within the optic nerve are associated with a greater mitochondrial load. The axons of the RGCs are unmyelinated within the retina, an adaptation which likely exists to prevent myelin from impeding light reaching the photoreceptors. Myelination initiates following the lamina cribrosa, a network of collagen fibers in the optic nerve head approximately 400 μm thick the RGC axons traverse to pass through the sclera. The unmyelinated region is associated with a higher density of mitochondria than the adjacent myelinated sections. This unique architecture of the RGCs is a possible explanation as to why the optic nerve appears so vulnerable to degeneration associated with mitochondrial dysfunction.

The loss of sight in autosomal OA is associated with the degeneration of retinal ganglion cells (RGCs), which transmit visual information from the photoreceptors to the brain (Miyata et al., 2007). Collectively, the axons of the RGCs form the optic nerve (Figure 1). As with most neurons, RGCs have a high energy demand to ensure the continuous active transport of ions against their concentration and electrical gradients required in membrane repolarization, maintenance of calcium stores and synaptic vesicle mobilization (Wong-Riley, 2010). This energy is provided primarily through electron transport within the cristae folds of the inner mitochondrial membrane (IMM) coupled to oxidative phosphorylation (OXPHOS) to synthesize ATP. Morphologically, RGCs comprise complex dendritic arbors and long axons. Moreover, and somewhat uniquely to RGCs, the most proximal portion of the axons, within the retina, are unmyelinated (Perry and Lund, 1990). The unmyelinated region of RGC axons have a higher mitochondrial load (Figure 1; Perge et al., 2009; Wilkison et al., 2021). This is generally believed to reflect an increased energy requirement to propagate axon potentials compared to myelinated axons, however recent findings show that mitochondrial accumulation precedes RGC axon myelination bringing this supposition into question (Wilkison et al., 2021). Furthermore, while RGCs seem to be particularly vulnerable to mitochondrial defects, autosomal OA-causing mutations can result in degeneration of neurons other than RGCs as evidenced by patients with syndromic-OA. Additionally, there are several reports of the co-occurrence of autosomal OA and other neurodegenerative disorders specifically hereditary spastic paraplegia (HSP) (Yu-Wai-Man et al., 2010b; Charif et al., 2020) and Charcot-Marie-Tooth type 2 (CMT2) (Rouzier et al., 2012; Guerriero et al., 2020). Together, these provide compelling evidence that highly elongated neurons, such as RGCs, motor neurons, sensory neurons and cerebella purkinje cells, are particularly sensitive to the mutations underpinning these neurodegenerative disorders. This emphasizes the importance of comparable *in vivo* models so that molecular events specifically within long axons may be better understood.

Autosomal OA is a genetically heterogeneous, monogenic disorder, primarily caused by mutations in nuclear genes encoding mitochondrial proteins. Mutations in *OPA1* are the most common cause of autosomal OA (Almind et al., 2012; Weisschuh et al., 2021). However, as discussed in detail below, mutations in at least 10 other genes are also associated with autosomal OA. Identified disease-causing mutations run the spectrum from nonsense, missense frameshift and splice mutations, but chromosomal rearrangements including copy number variants and inversions were also identified (Fuhrmann et al., 2009; Weisschuh et al., 2021). The majority of disease-causing mutations are predicted to impair the function of the encoded protein, pointing to haploinsuffiency as the primary mechanism of pathogenicity in dominant forms of autosomal OA (Neumann et al., 2020; Sun et al., 2021). However, several cases report semi-dominant inheritance, where individuals carrying more than one OA-causing mutation present with much more severe disease than heterozygotic parents or siblings (Pesch et al., 2001; Weisschuh et al., 2021). Several forms of recessively inherited OA have also been identified (associated with mutations in *TMEM126A/OPA7*, *SCL25A46*, *MCAT* and *RTN4IP1/OPA10*) and mutations in *WFS1*, *ACO2/OPA9* and *OPA3* are associated with both recessive and dominant forms of OA (Reynier et al., 2004; Meyer et al., 2010; Kloth et al., 2019; Charif et al., 2021b). Given this genetic heterogeneity, we use the term ‘autosomal OA’ throughout this review.

**FIGURE 2:**
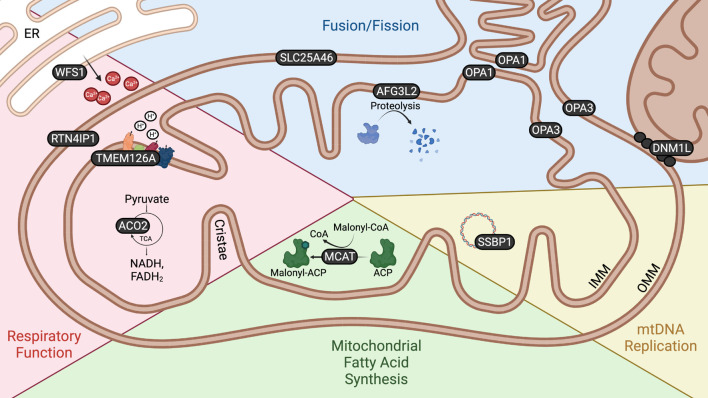
Mitochondrial localization and function of autosomal OA genes. Schematic demonstrating the localization and function of proteins encoded by autosomal OA-associated genes. The localization of proteins encoded by autosomal OA-associated genes within and in association with the mitochondria emphasizes the relevance of these organelles to autosomal OA. WFS1 is localized to the ER, but is enriched at MAMs. DNM1L is recruited from the cytoplasm to the OMM to facilitate mitochondrial fission. There is conflicting evidence as to whether OPA3 is localized to the IMM or OMM.

All of the genes implicated in autosomal OA encode proteins associated with mitochondrial function, the majority being nuclear genes encoding mitochondrial proteins (Charif et al., 2021a). These genes have roles in mitochondrial fission or fusion, mitochondrial respiration, mitochondrial DNA replication and mitochondrial fatty acid synthesis (Figure 2). Furthermore, disrupted mitochondrial network morphology and reduced respiratory efficiency are consistently observed in autosomal OA patient fibroblasts (Kane et al., 2017; Liao et al., 2017) as well as in cell and animal models (Rahn et al., 2013; Maloney et al., 2020). Autosomal OA is not unique amongst optic neuropathies to be characterized by mitochondrial dysfunction. Mitochondrial dysfunction is a common feature of many retinal diseases including: Leber’s Hereditary Optic Neuropathy (LHON) and glaucoma (Bahr et al., 2020; Duarte, 2021). Indeed, mitochondrial dysfunction is now recognized as a common feature of axonopathies generally (Krols et al., 2016). While there is strong evidence to suggest mitochondrial dysfunction is central to neurodegeneration in autosomal OA, the underlying mechanism, and why RGCs are particularly vulnerable, remains unknown. In this article we provide a comprehensive review of studies investigating the functions of known autosomal OA-causing genes and emphasize that, despite their heterogeneity, there are just a few converging pathogenic themes emerging. Furthermore, we investigate the range of *in vivo* models available which offer potential to better understand the molecular and cellular events underpinning autosomal OA, and assess their suitability to uncover why RGCs may be particularly targeted in OA and to identify novel therapeutic strategies.

## Gene Mutations Causing Autosomal Optic Atrophy

Most currently identified genes causative of autosomal OA can be broadly classified into two functional categories; mitochondrial fission/fusion and mitochondrial respiration; albeit two autosomal OA genes, with roles in mtDNA replication and mitochondrial fatty acid synthesis, do not clearly fit into either of these categories. These categories are not mutually exclusive; on the contrary, disruption of mitochondrial morphology through altered fission/fusion dynamics is known to alter mitochondrial respiration and mtDNA stability (Chen et al., 2010; Quintana-Cabrera et al., 2018; Glancy et al., 2020). Below we examine the function of proteins encoded by OA-causing genes and the mitochondrial aberrations associated with disease-causing mutations. For this review, we focus on autosomal OA-causing genes linked to more than two families where there is clear evidence that OA is the primary feature of disease (Table 1). We therefore do not include discussion on genes that cause optic atrophy as a feature of another neurological disorder such as *NDUFS2* (primarily associated with Leigh syndrome) (Gerber et al., 2017a), *SPG7* (primarily associated with HSP) (Charif et al., 2020), CISD2 (primarily associated with Wolfram syndrome) (Delprat et al., 2018), C19orf12 (primarily associated with MPAN syndrome) (Mignani et al., 2020) or MFN2 (primarily associated with CMT2) (Bombelli et al., 2014). The role of many of these genes in mitochondrial function has been recently reviewed elsewhere (Maresca and Carelli, 2021).

**TABLE 1:** Summary of autosomal OA-associated genes and their inheritance.

Gene	Inheritance	Disease presentation	Reported Mitochondrial Dysfunction
*OPA1*	Mainly dominant	Mainly non-syndromic	Fragmented mitochondrial network, abnormal cristae, respiratory defects, mtDNA depletion
*AFG3L2/OPA12*	Mainly dominant	Mainly non-syndromic	Increased proteolytic processing of OPA1, mitochondrial fragmentation
*SLC25A46*	Recessive	Syndromic	Abnormal mitochondrial morphology, abnormal cristae, depletion of the MICOS complex, respiratory defects, mtDNA maintenance deficit
*OPA3*	Mainly dominant	Mainly non-syndromic	Elongated mitochondria, abnormal cristae, aberrant respiratory function
*DNM1L/OPA5*	Dominant	Non-syndromic	Elongated mitochondria, reduced mitochondria/peroxisome turnover, reduced respiratory capacity
*WFS1*	Mainly recessive	Both syndromic and non-syndromic	Reduced mitochondria-ER contacts, mitochondrial calcium accumulation, loss of mitochondrial membrane potential, reduced ATP production
*ACO2/OPA9*	Both	Mainly non-syndromic	Respiratory defects, impaired mtDNA maintenance
*RTN4IP1/OPA10*	Recessive	Syndromic	Fragmented mitochondria, respiratory defects
*TMEM126A/OPA7*	Recessive	Mainly non-syndromic	Respiratory defects
*SSBP1/OPA13*	Dominant	Mainly non-syndromic	mtDNA depletion, abnormal cristae, respiratory deficit
*MCAT*	Recessive	Non-syndromic	Respiratory Defects, abnormal mitochondrial morphology

### Mitochondrial Fission/Fusion

Mitochondria are highly dynamic organelles which constantly undergo fission, whereby mitochondria divide into smaller ‘daughter’ mitochondria, and fusion, whereby two or more mitochondria fuse together. These processes permit mitochondria to regulate their distribution according to metabolic demand (Wai and Langer, 2016), to communicate mtDNA and protein content (Silva Ramos et al., 2016) and to regulate mitochondrial turnover via mitophagy (Xian and Liou, 2021). To date, five genes encoding proteins which function in mitochondrial fission/fusion have been associated with autosomal OA: *OPA1*, *AFG3L2*/*OPA12*; *SLC25A46*; *OPA3*; *DNM1L*/*OPA5* (Figure 2).

### OPA1

*Optic atrophy 1 (OPA1)* is a ubiquitously expressed, highly conserved gene with *OPA1* homologs present in all higher metazoans (Wong et al., 2003; Li et al., 2019). It encodes a dynamin-like GTPase localized to the inner mitochondrial membrane (IMM) and is essential for IMM fusion following the fusion of the outer mitochondrial membrane (OMM) by the mitofusins (MFN1 and MFN2) (Song et al., 2009). OPA1 is produced as a long-form (L-OPA1) which can be cleaved by either of two proteolytic enzymes, YME1L or OMA1 respectively, to produce short-form OPA1 (S-OPA1) (Anand et al., 2014). Regulation of mitochondrial fusion depends on OPA1 processing and while either L- or S-OPA1 are sufficient to induce mitochondrial fusion, optimal fusion requires the presence of equimolar concentrations of both isoforms (Anand et al., 2014; Ge et al., 2020; Ohba et al., 2020). *OPA1* is the most common gene implicated in autosomal OA, accounting for roughly 70% of cases (Ferré et al., 2009). Non-syndromic-autosomal OA appears to be generally associated with mutations resulting in haploinsufficiency, whilst missense mutations are associated with syndromic-autosomal OA, and more severe ocular symptoms (Carelli et al., 2015; Ham et al., 2019). This is proposed to be due to a dominant negative effect (Amati-Bonneau et al., 2009). Approximately 20% of *OPA1* patients have extra ocular symptoms, most commonly sensorineural deafness, but additionally peripheral neuropathy, ataxia, myopathy and parkinsonism (Lynch et al., 2017; Ham et al., 2019). Although the vast majority of *OPA1* mutations are inherited in an autosomal dominant manner, there are examples of individuals carrying 2 apparently recessive pathogenic *OPA1* mutations, usually exhibiting compound heterozygosity. These patients present with a more severe syndromic-autosomal OA, often associated with unusual features such as developmental delays, pyramidal tract abnormalities, and neuromuscular disorders (Zerem et al., 2019; Zeng et al., 2020). Some *OPA1* mutations also appear to exhibit incomplete penetrance, with many examples of seemingly unaffected carriers of pathogenic mutations, as well as variable pathology within families bearing the same mutation (Toomes et al., 2001; Cohn et al., 2008). Whilst no modifying genes have been identified to explain this, there is evidence to suggest alcohol and tobacco consumption may be associated with more severe symptoms (Mei et al., 2019). Both patient fibroblasts and cell models bearing pathogenic mutations of *OPA1* have a characteristic fragmented mitochondrial network, consistent with a mitochondrial fusion disorder, and highly disordered cristae (Liao et al., 2017; Del Dotto et al., 2018). Through its key role in cristae organization, changes in OPA1 expression affect the stability of the respiratory supercomplexes (Cogliati et al., 2013), quaternary structures adopted by components of the electron transport chain to carry out cellular respiration (Letts et al., 2016). Subsequently, defects in OXPHOS are often associated with insufficient OPA1 activity (Lee et al., 2017; Sun et al., 2020). Consistent with this, widespread respiratory defects, including decreased ATP synthesis, increased oxygen consumption and reduced respiration, have all been reported in *OPA1* patient fibroblasts and cell models (Zanna et al., 2008; Millet et al., 2016; Zhang et al., 2017). The manner and extent to which respiration is affected appears to vary between *OPA1* mutations, with some evidence to suggest that respiratory defects are associated with more severe vision loss (van Bergen et al., 2011). It is perhaps unsurprising that there is substantial variation between patients, due to incomplete penetrance and heterogeneity of symptoms.

### AFG3L2/OPA12

*AFG3L2/OPA12* encodes a mitochondrial AAA quality-control protease which is tethered to the IMM and exposes its enzymatic domain to the mitochondrial matrix (Puchades et al., 2019). Missense mutations, generally within the AAA domain of *AFG3L2/OPA12*, give rise to isolated, dominantly inherited autosomal OA, though rarer biallelic mutations have been reported which result in more severe, syndromic-autosomal OA (Colavito et al., 2017; Baderna et al., 2020; Caporali et al., 2020). Of note, missense mutations in *AFG3L2/OPA12* are also known to cause the unrelated disorders spinocerebellar ataxia type 28 (SCA28) and spastic ataxia syndrome 5 (SPAX5) but these mutations are generally localized within the proteolytic domain of the protein (Caporali et al., 2020). AFG3L2 is known to have vital roles in mitochondrial maintenance including mitochondrial ribosome assembly, electron transport chain complex processing and calcium homeostasis (Puchades et al., 2019; Pareek and Pallanck, 2020) however, the mechanism by which missense mutations give rise to autosomal OA was only recently discovered. Two independent studies revealed that autosomal OA-causing mutations in *AFG3L2/OPA12* cause hyperactivation of OMA1, the protease that acts on L-OPA1, resulting in increased processing of L-OPA1 to S-OPA1 (Baderna et al., 2020; Caporali et al., 2020). This imbalance in L-OPA1/S-OPA1 results in mitochondrial fragmentation which is evident in both engineered MEFs and patient fibroblasts.

### SLC25A46

*SLC25A46* encodes a member of the solute carrier family 25 (SLC25), a group of proteins primarily localized to the IMM containing six alpha-helical membrane spanning domains (Pebay-Peyroula et al., 2003). Many members of the SLC25 family are responsible for the transport of metabolic intermediates (such as glutamate and ADP/ATP) into the cell (Fiermonte et al., 2002; Kunji et al., 2016), with some functioning as uncoupling proteins (Fedorenko et al., 2012). SLC25A46 is unusual in the SLC25 protein family in that it localizes to the OMM (Janer et al., 2016) and has no known transporter function nor any identified substrates. Recessive, loss-of-function mutations in *SLC25A46* are associated with syndromic-autosomal OA (Nguyen et al., 2017; Bitetto et al., 2020). There are a wide spectrum of reported symptoms, including Parkinsonism, Leigh syndrome, cerebellar ataxia, and congenital pontocerebellar hypoplasia, usually accompanied by optic atrophy (Wan et al., 2016; Hammer et al., 2017; Bitetto et al., 2020). Whilst the exact function of SLC25A46 is unknown, it likely has a role in mitochondrial fission/fusion dynamics. Loss of SLC25A46 function has been associated with both elongated mitochondria, suggestive of aberrant fission (Abrams et al., 2015; Wan et al., 2016) and small, circular mitochondria, which would be indicative of a fusion defect (Duchesne et al., 2017). Overexpression appears to induce fragmentation in the mitochondrial network, which would also be consistent with a role in fission (Abrams et al., 2015). Both patient samples and SLC25A46 models usually display highly abnormal mitochondrial cristae (Janer et al., 2016; Li et al., 2017; Zou et al., 2021) in some cases being completely detached from the IMM (Duchesne et al., 2017). Consistent with this, SLC25A46 is thought to operate upstream of the MICOS complex, which is significantly depleted in *SLC25A46* knockout cells. The MICOS is a large protein complex localized to the IMM where it is crucial for the structural organization of the cristae (Friedman et al., 2015) and interacts with both OPA1 and MFN2 (Janer et al., 2016). Depletion of its constituents have been shown to have deleterious effects on processes reliant on the structural integrity of the cristae, such as mtDNA maintenance and respiration (John et al., 2005; Genin et al., 2016; Gödiker et al., 2018).

### OPA3

*OPA3* encodes a protein of unknown function localized to mitochondria, with conflicting evidence as to whether it localizes to the IMM or OMM (Da Cruz et al., 2003; Ryu et al., 2010). Most autosomal OA-causing mutations in *OPA3* are dominantly inherited and result in non-syndromic disease, although hearing loss and extra-ocular neurological symptoms consistent with syndromic-autosomal OA have also been observed in some patients (Reynier et al., 2004; Sergouniotis et al., 2015). Recessive loss of function *OPA3* mutations are most often associated with Costeff syndrome, which resembles syndromic-autosomal OA with some additional features such as cognitive deficit and extrapyramidal dysfunction (Grau et al., 2013). While there remains ambiguity surrounding the precise function of OPA3, overexpression and knockout studies support a role in mitochondrial fission/fusion dynamics. Overexpression of OPA3 in patient-derived or retinal pigment epithelium-cell models creates a highly fragmented mitochondrial network, suggestive of a role in mitochondrial fission (Ryu et al., 2010; Maresca et al., 2012). Consistent with this, fibroblasts from some *OPA3* patients were recently reported to have enlarged mitochondria with slightly fragmented cristae (Horga et al., 2019). It is not currently clear whether the altered mitochondrial morphology resulting from *OPA3* mutations disrupts mitochondrial function with conflicting reports on the effect of loss of OPA3 on ATP production and oxygen consumption (Pei et al., 2010; Meng et al., 2020).

### DNM1L/OPA5

*Dynamin 1-like (DNM1L)/OPA5* encodes a dynamin-like GTPase which is an essential protein in the regulation of mitochondrial fission. DNM1L is recruited to the OMM from the cytosol by mitochondrial fission factors: MFF, MID49, and MID51, where it oligomerizes forming a ring-like structure around the mitochondria (Palmer et al., 2011; Fröhlich et al., 2013). It then uses GTP hydrolysis to constrict the membrane, which can subsequently undergo scission. Presently, there are very few examples of autosomal OA patients with *DNM1L/OPA5* variations (Gerber et al., 2017b). These patients, from three families, harbor 2 separate mutations within the GTPase domain, and appear to exhibit dominant inheritance. All patients present with non-syndromic-autosomal OA, except one individual who additionally had slight hearing loss. *DNM1L/OPA5* mutations have previously been associated with forms of severe encephalopathy and epilepsy, often associated with infant mortality (Fahrner et al., 2016; Zaha et al., 2016). These disease-causing mutations have been reported in both the middle and GTPase domains of DNM1L and, apart from rare examples (Waterham et al., 2007; Chao et al., 2016), do not appear to be associated with optic atrophy (Fahrner et al., 2016; Ladds et al., 2018). Patient fibroblasts from individuals with non-syndromic-autosomal OA, as well as a yeast model expressing the patient mutation, display an elongated mitochondrial network, consistent with a mitochondrial fission defect (Gerber et al., 2017b). However, alterations in respiratory capacity, aberrant peroxisomes or reduced mitochondrial/peroxisomal turnover, which have been reported in samples from DNM1L-mediated encephalopathy patients, are not observed models of OA (Sheffer et al., 2016; Zaha et al., 2016; Longo et al., 2020).

Three further genes that function in the regulation of mitochondrial fission/fusion have recently been associated with autosomal OA. These include the genes encoding the IMM protease *YME1L1/OPA11* and the OMM proteins mitochondrial elongation factor 1 (*MIEF1*) and mitochondrial fission factor (*MFF*). Only a few individuals or single families have been identified with these mutations thus far, hence did not fulfill our criteria for more extensive discussion, however it appears that heterozygous mutations in *MIEF1* cause dominantly inherited non-syndromic-autosomal OA (Charif et al., 2021c) while homozygous mutations in *YME1L1/OPA11* or *MFF* cause recessively inherited syndromic-autosomal OA (Shamseldin et al., 2012; Hartmann et al., 2016; Koch et al., 2016). All 3 of the proteins encoded by these genes function in the regulation of mitochondrial fission/fusion; YME1L1 via the proteolytic processing of OPA1 (Song et al., 2007; Stiburek et al., 2012), MIEF1 via the sequestration of DNM1L (Zhao et al., 2011; Yu et al., 2017) and MFF via recruitment of DNM1L to mitochondrial fission sites (Otera et al., 2010; Losón et al., 2013). Mitochondria in cells expressing disease-causing mutations or patient fibroblasts all display disrupted organization, consistent with defective regulation of fission/fusion (Koch et al., 2016; Cesnekova et al., 2018; MacVicar et al., 2019).

### Mitochondrial Respiratory Function

Respiration refers to a series of reactions through which the cell breaks down macronutrients to release their energy in the form of adenosine triphosphate (ATP). Mitochondria are essential for this process, housing both the citric acid cycle and electron transport chain. The citric acid cycle is a process through which the metabolic intermediate pyruvate undergoes a series of redox reactions to reduce the electron carriers NADH and FADH_2_, which then pass their electrons onto the respiratory complexes of the electron transport chain, ultimately producing most of the cell’s energy through OXPHOS (Papa et al., 2012). Neurons are usually highly energetically demanding, partly due to the process of neurotransmission itself (Harris et al., 2012), but additionally due to the comparatively large distance over which cargo, including organelles and synaptic components, must be transported along axons and dendrites (Mandal and Drerup, 2019). RGCs are no exception to this, in fact they are particularly vulnerable to bioenergetic perturbation. There is evidence that RGCs may utilize glycolysis in addition to OXPHOS to meet the considerable energetic demands imposed by their unique architecture (Trevisiol et al., 2017; Casson et al., 2021). Four autosomal OA-causing genes are associated with cellular respiration: *WSF1*, *ACO2*, *RTN4IP1* and *TMEM126A* (Figure 2).

### WFS1

After *OPA1*, the second most common gene associated with optic atrophy is wolframin (*WFS1*). Mutations in *WFS1* were primarily identified to cause Wolfram syndrome type 1 (WS1), a recessive condition characterized by diabetes, optic atrophy and deafness (Inoue et al., 1998; Wragg et al., 2018). However, mutations in *WFS1* are recognized as a frequent cause of optic atrophy independent of other WS1 symptoms (Hogewind et al., 2010; Grenier et al., 2016; Charif et al., 2021a). In fact, a recent screen of over 1,000 autosomal OA patients identified *WFS1* mutations in 12% of dominant-autosomal OA and 39% of receive-autosomal OA patients (Charif et al., 2021a). Autosomal OA mutations are most commonly missense mutations, though truncating mutations have been identified (Hogewind et al., 2010; Grenier et al., 2016). A reduction in available protein is proposed as the pathogenic mechanism, with missense mutations appearing to decrease the half-life of the resulting wolframin protein (Hofmann et al., 2003). This was supported by a recent study which found that the extent of optic atrophy progression in patients correlates with the degree of decrease in wolframin protein (Hu et al., 2021). Wolframin is a transmembrane protein enriched at mitochondria-associated ER membranes (MAM) (La Morgia et al., 2020). It is ubiquitously expressed with particularly high levels of expression in the optic-nerve (Yamamoto et al., 2006; Schmidt-Kastner et al., 2009). Wolframin has roles in many cellular pathways including ER stress (Fonseca et al., 2010; Odisho et al., 2015), calcium homeostasis (Takei et al., 2006; Nguyen et al., 2020) and mitochondrial activity (Kõks et al., 2013; Angebault et al., 2018). However, there exists good evidence that disruption of mitochondrial function by loss of WFS1 is critical to pathogenicity in OA. Mitochondrial metabolism and ATP production are tightly regulated by the proper transfer of calcium ions from the ER at MAMs (Denton, 2009; Llorente-Folch et al., 2015) and disruption of MAMs impacts mitochondrial function in several models of neurodegenerative disease (Lee et al., 2018). Wolframin localizes to MAMs and the proportion of mitochondria in contact with the ER is significantly reduced in fibroblasts from patients carrying *WFS1* mutations (Angebault et al., 2018; La Morgia et al., 2020). As predicted, this MAM disruption results in reduced calcium accumulation within mitochondria and decreased mitochondrial function in patient fibroblasts (Angebault et al., 2018; La Morgia et al., 2020). Moreover, primary cortical neurons in which WFS1 has been knocked down by siRNA display decreased membrane potential and cytosolic ATP (Cagalinec et al., 2016) further supporting a role for mitochondrial dysfunction underpinning neurodegeneration in autosomal OA caused by *WFS1* mutations.

### ACO2/OPA9

Mutations in the gene encoding citric acid cycle enzyme aconitase 2 (*ACO2*)/*OPA9* were until recently believed to be quite a rare cause of inherited optic atrophy. Individual families had been identified with loss-of-function *ACO2* mutations leading to both dominantly inherited syndromic-autosomal OA or recessively inherited non-syndromic-autosomal OA (Metodiev et al., 2014; Kelman et al., 2018; Neumann et al., 2020). However, a recent study using next-generation sequencing to identify causative genes in a large, multicenter cohort of patients with autosomal optic neuropathies, reported that 12% of autosomal OA cases had pathogenic mutations in the gene *ACO2* (Charif et al., 2021b). In this study, recessive cases displayed significantly worse vision loss than dominant cases while 12–27% displayed extraocular symptoms (Charif et al., 2021b). This implies that *ACO2/OPA9* mutations are the third leading cause of autosomal OA after *OPA1* and *WFS1*. *ACO2/OPA9* encodes the mitochondrial aconitase 2 enzyme, a lyase that converts citrate to isocitrate within the citric acid cycle (Martius, 1937). The citric acid cycle consists of a series of redox reactions in the mitochondrial matrix through which pyruvate, the final product of glycolysis, is used to reduce the electron carriers NAD and FADH for the OXPHOS pathway. Through this mechanism, ACO2 is important for generating ATP through aerobic respiration. To date, disease-causing mutations have not been studied in neuronal cells, but patient-derived fibroblast consistently show reduced ACO2 enzymatic activity, impaired maintenance of mtDNA and decreased respiratory function (Neumann et al., 2020; Charif et al., 2021b). This supports the hypothesis that mitochondrial dysfunction underpins RGC degeneration in these patients, though animal models to validate and study this further are required.

### RTN4IP1/OPA10

*Reticulon 4 interacting protein 1 (RTN4IP1)/OPA10* encodes a widely expressed protein localized to the OMM (Hu et al., 2002; Angebault et al., 2015). It contains an oxidoreductase domain and is believed to act on Complex I of the mitochondrial respiratory chain (Hu et al., 2002). Homozygous or compound heterozygous mutations in *RTN4IP1/OPA10* are found across many different populations to cause recessive forms of autosomal OA (Okamoto et al., 2017; Zou et al., 2019; D’Gama et al., 2021). Patients generally present with syndromic autosomal OA, with epilepsy, chorea and encephalopathy commonly reported, and it is suggested that more severe symptoms are associated with more deleterious mutations in the *RTN4IP1/OPA10* gene (D’Gama et al., 2021). Recently, it has also been reported that some patients experience degeneration of the rods in addition to RGCs, expanding the spectrum of neurons that are disrupted by loss of this protein (Meunier et al., 2021; Rajabian et al., 2021). Autosomal OA-causing mutations result in a marked decrease or loss of RTN4IP1 protein expression in patient fibroblasts and muscle biopsies (Angebault et al., 2015; Charif et al., 2017). Moreover, there is clear evidence that mitochondrial function are disrupted by these mutations. Examination of patient fibroblasts reveals a reduction in activity of the mitochondrial respiratory chain, specifically Complex I (Angebault et al., 2015; Charif et al., 2017). Whilst the precise function of RTN4IP1 is currently unknown, Angebault et al. (2015) generated both mouse and zebrafish models with depleted RTN4IP1. Mouse pups demonstrated an increase in dendrite numbers and dendrite arborization, whilst the RGC layer was completely absent from the retina of zebrafish RTN4IP1 morphants. Whilst human samples were not available for histological analysis, patients did appear to exhibit smaller optic disks. This implies that RTN4IP1 has an important developmental role linked to the RGCs which may contribute to OA pathogenesis.

### TMEM126A/OPA7

*TMEM126A/OPA7* encodes a highly conserved IMM protein which functions in the mitochondrial respiratory chain Complex I assembly pathway. Homozygous mutations, both missense and nonsense mutations, are reported to cause recessive forms of autosomal OA (Hanein et al., 2009; Meyer et al., 2010; Li J. K. et al., 2020). Patients primarily present with non-syndromic autosomal OA, but auditory neuropathy has been reported in some families (Meyer et al., 2010; Désir et al., 2012; La Morgia et al., 2019). Recently, two independent studies show that TMEM126A is necessary for the biogenesis and function of mitochondrial respiratory chain Complex I (D’Angelo et al., 2021; Formosa et al., 2021). *TMEM126A/OPA7* knockout cells are deficient for Complex I and display decreased respiratory capacity (D’Angelo et al., 2021; Formosa et al., 2021). Significantly, autosomal OA patients with *TMEM126A/OPA7* mutations show partial Complex I deficiency in addition to increased lactic acid levels following exercise (Hanein et al., 2009; La Morgia et al., 2019), suggesting that defective OXPHOS contributes to disease pathogenesis in these patients.

### Mitochondrial Fatty Acid Synthesis and mtDNA Replication

Currently, two autosomal OA-causing genes do not clearly fit into the functions contributing to mitochondrial fission/fusion or respiratory function, specifically *SSBP1* and *MCAT* which function in the regulation of mitochondrial DNA replication and mitochondrial fatty acid synthesis, respectively. While these genes may appear to be disparate to other OA-causing genes in terms of their roles in mitochondria, there are clear similarities in the mitochondrial dysfunction observed when these genes are disrupted. Specifically mitochondrial morphology and respiratory function are impaired in patient fibroblasts or knockout cells, suggesting that the underlying pathogenic mechanism of action may be analogous to that of other OA-causing genes. Furthermore, with a growing list of novel OA-causing mutations being identified, it may be that other genes with similar functions are identified to cause OA over the coming years.

### SSBP1/OPA13

*Single-stranded DNA binding protein 1 (SSBP1)/OPA13* is a nuclear-encoded housekeeping gene involved in mitochondrial biogenesis. *SSBP1/OPA13* encodes a constituent of the mtDNA replisome which, along with the mitochondrial DNA polymerase Pol γ and the helicase Twinkle, is one of the essential components for mtDNA replication (Korhonen et al., 2004). During mtDNA synthesis, SSBP1 binds the heavy strand, increasing the activity of Pol γ through maintaining the ssDNA in a favorable conformation and preventing the formation of secondary structure (Ciesielski et al., 2015). SSBP1 also stimulates the unwinding activity of Twinkle (Oliveira and Kaguni, 2011). Missense mutations in *SSBP1/OPA13* cause autosomal OA in at least 14 different European families, though the prevalence of this mutation in other populations is not known (Jurkute et al., 2019; Del Dotto et al., 2020; Piro-Megy et al., 2020). Almost all families show dominant inheritance of non-syndromic autosomal OA and rod-cone dystrophy in addition to the optic atrophy is common, indicating that retinal neurons other than RGCs are susceptible to mitochondrial dysfunction induced by *SSBP1/OPA13* mutations (Jurkute et al., 2019; Del Dotto et al., 2020). Autosomal OA-causing mutations in *SSBP1/OPA13* do not affect gene expression, though stability of the SSBP1 protein, particularly protein dimers, is likely reduced by some missense mutations (Del Dotto et al., 2020; Piro-Megy et al., 2020). Moreover, patient fibroblasts consistently reveal significant depletion of mtDNA, providing strong evidence that autosomal OA-causing mutations destabilize the replication machinery and disrupt the fidelity of mtDNA replication (Del Dotto et al., 2020; Piro-Megy et al., 2020). Loss of mtDNA results in swollen mitochondria with disorganized cristae and a severe respiratory deficit, which are common in patient fibroblasts (Del Dotto et al., 2020; Piro-Megy et al., 2020). This is consistent with mitochondrial disruption observed in SSBP1 knockdown cells (Wang et al., 2017) and supports a loss of function pathogenic mechanism underpinning autosomal OA in these patients.

### MCAT

*Malonyl-CoA-acyl carrier protein transacylase (MCAT)* is a nuclear-encoded gene involved in mitochondrial fatty acid synthesis (mtFAS), a comparatively poorly understood pathway distinct from the cytosolic equivalent. Malonyl-CoA is a key intermediate in mtFAS, transferring malonate from malonyl-CoA to the acyl carrier protein (ACP), an important step in fatty acid elongation. A rare cause of autosomal OA, *MCAT* mutations give rise to non-syndromic-autosomal OA, and appear to have a recessive mode of inheritance (Li H. et al., 2020; Gerber et al., 2021). *In silico* analysis suggests autosomal OA-causing mutations make the resulting MCAT protein unstable and less able to bind malonyl-CoA (Gerber et al., 2021), however, this has not been examined directly in patient samples or model systems. Studies in conditional *Mcat* knockout mice reveal that the mtFAS pathway produces the octanoyl precursor required for lipoylation of key mitochondrial proteins involved in the citric acid cycle, namely pyruvate dehydrogenase complex and α-ketoglutarate dehydrogenase (Smith et al., 2012). Depletion of *Mcat*, or other genes involved in mtFAS, results in a significant depletion of the respiratory complexes and limits the ability of mitochondria to respire (Smith et al., 2012; Nowinski et al., 2020). Mitochondria in cells expressing *MCAT* mutations are disrupted, displaying a thin, thread-like morphology with unidentified debris within the mitochondrial matrix (Li H. et al., 2020). Moreover, they show a reduced ability to uptake MitoTracker, consistent with impaired IMM function, specifically an inability to sustain its membrane potential (Keij et al., 2000). Interestingly, mutations in another mtFAS component, *MECR*, cause the rare neurological disorder MEPAN syndrome, which features optic atrophy (Heimer et al., 2016; Gorukmez et al., 2019). Further study is needed to characterize this pathway, in order to better understand why perturbation leads to mitochondrial dysfunction.

Taken together, it is clear that there are converging pathogenic pathways related to mitochondrial organization and function underpinning autosomally inherited OA. Going forward, we should look to studies of animal models of OA to help elucidate the particular susceptibility of RGCs and other long axons to these disruptions, as well as to identify promising targets for therapeutic interventions.

## *In vivo* Models of Autosomal Optic Atrophy

Whilst *in vitro* models are invaluable for investigating the molecular underpinnings of disease, there are limitations in their applicability to studying OA. The specific architecture of RGCs, with long and heterogeneously myelinated axons, cannot yet be accurately replicated in cell culture nor in non-polarized cells such as patient fibroblasts. Moreover, as the progressive loss of visual acuity is a key unifying symptom of OA, the ability to assess visual function is paramount to both the study of OA disease pathology and assessing the value of any potential treatment. This necessitates the use of *in vivo* models which can be used for *in situ* examination of RGCs and optic nerves, as well as functional studies reflecting changes in visual function in response to OA-associated genetic mutations, and subsequently assessing treatments. This section evaluates the utility of the most commonly used animal models in the study of mitochondrial disruption in neurons of relevance to OA.

### Mouse Models

Anatomically, the mouse retina is stratified in a very similar manner to that of humans, with the outermost ganglion cell layer facing the vitreous body and the innermost retinal pigment epithelium adjacent to the choroid. There are however some notable differences, for example, mice do not possess a collagenous lamina cribrosa, which is relevant to the study of OA as it surrounds and supports the unmyelinated RGC axons as they exit the sclera and form the optic nerve (Chen et al., 2017). Optic nerve density also appears to vary considerably between populations of laboratory mice, which may affect susceptibility to optic nerve atrophy, and should be considered when selecting backgrounds for autosomal OA models (Jeon et al., 1998). As with all models – gene editing – in particular CRISPR/Cas9 mediated gene editing, has become an invaluable tool for recapitulating genetic disease in mice. There are many established protocols for creating KOs, recreating patient-specific mutations and the insertion of human genes of interest into mice (Platt et al., 2014; Chen et al., 2019; Min et al., 2019). However, there are important considerations when selecting a laboratory strain to develop an autosomal OA model as several strains of laboratory mice develop age-related hearing loss, a common extra-ocular symptom of many autosomal OAs (Turner et al., 2005), and basal visual acuity varies significantly between strains (Wong and Brown, 2006), which may confound results. An advantage of mice for modeling OA is the array of assays available for examining both visual function and RGC morphology. Typically, measuring visual acuity orientates around measuring the optokinetic and optomotor reflexes and the pupillary response (Kretschmer et al., 2017). A significant obstacle is that some of these assays require some form of trained behavior. Mice are proficient learners at repetitive tasks, therefore increased task performance over time may somewhat obscure aspects of the degenerative phenotype seen in most forms of OA (Birtalan et al., 2020).

Many mouse models of autosomal OA have been reported and several common features are apparent from these studies (Table 2). Dominantly inherited autosomal OA-causing mutations are generally found to be embryonic lethal in homozygous mice and as such, many mouse studies linked to autosomal OA are conducted in heterozygotes (Wakabayashi et al., 2009; Jiang et al., 2021). Some studies report a progressive loss of visual acuity (Davies et al., 2007; Zaninello et al., 2020), although most do not investigate the presence of a visual phenotype. Many studies observe progressive degeneration of the RGCs which in some cases is accompanied by myelination defects of the optic and peripheral nerves (Li et al., 2017; Waszczykowska et al., 2020). Most models exhibit extra-ocular symptoms common to syndromic-autosomal OA, including progressive motor and hearing impairments often attributed to neuropathy (Sarzi et al., 2012; Mancini et al., 2019). Significantly, nearly all autosomal OA mouse models show some form of neuronal mitochondrial dysfunction, generally in the form of a highly fragmented mitochondrial network with a reduced number of malformed cristae (Powell et al., 2011; Sarzi et al., 2012). Elongated networks have also been observed in mice with *Slc25A46* and *Dnm1l* mutations, consistent with observations from patient fibroblasts (Wan et al., 2016; Gerber et al., 2017b). Furthermore, several mouse models faithfully recapitulate gene-specific symptoms, such as glucose intolerance in *Wfs1* mutants (Ishihara et al., 2004; Kõks et al., 2009). Taken together, it’s clear that mouse models of autosomal OA can recapitulate key characteristics of this disorder. Despite many autosomal OA-type phenotypes, there are several phenotypes which are consistently observed in mouse autosomal OA-models which are not generally associated with the corresponding human pathogenic mutations. Most commonly, mice with loss of autosomal OA-causing genes display: reduced body size and difficulty gaining weight (Smith et al., 2012; Wells et al., 2012) and cardiomyopathy (Davies et al., 2008; Jiang et al., 2021). In many cases, these symptoms are not detectable in patients carrying the comparable pathogenic mutation and whilst many autosomal OA-causing genes are thought to have a developmental role in humans, there is presently little evidence for developmental abnormalities affecting these organs in patients (Maltecca et al., 2008; Caglayan et al., 2020).

**TABLE 2:** Summary of phenotypes observed in *in vivo* models of autosomal OA.

Phenotypes observed	Mouse 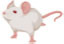	Zebrafish 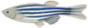	Fruit fly 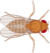	Worm 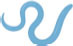
**Homozygous lethal**	***Opa1*** *(Davies et al., 2008)*, ***Ssbp1*** *(Jiang et al., 2021), **Dnm1l** (Wakabayashi et al., 2009)*	***Wfs1*** *(Cairns, 2019)*	***Opa1*** *(Shahrestani et al., 2009)*, ***Dnm1l*** *(Batzir et al., 2019)*, ***Ssbp1*** *(Maier et al., 2001)*, ***Afg3l2*** *(Pareek and Pallanck, 2020)*	***Aco2*** *(Simmer et al., 2003)*, ***Afg3l2*** *(Zubovych et al., 2010)*, ***Dnm1l*** *(Breckenridge et al., 2008)*
**Reduced lifespan**	***Opa3*** *(Davies et al., 2008)*, ***Afg3l2*** *(Maltecca et al., 2008)*, ***Mcat*** *(Smith et al., 2012)*	***Opa1*** *(Rahn et al., 2013)*, ***Opa3*** *(Pei et al., 2010)*	***Opa1** (Tang et al., 2009), **Wfs1** (Sakakibara et al., 2018), **Afg3l2** (Pareek and Pallanck, 2020)*	***Opa1** (Byrne et al., 2019)*
**Abnormal mitochondria**	***Opa1*** *(Davies et al., 2008)*, ***Sspb1*** *(Jiang et al., 2021)*, ***Dnm1l*** *(Gerber et al., 2017b)*, ***Opa3*** *(Powell et al., 2011)*, ***Afg3l2*** *(Mancini et al., 2019)*, ***Mcat*** *(Smith et al., 2012)*	***Opa1*** *(Eijkenboom et al., 2019)*, ***Wfs1*** *(Cairns, 2019)*, ***Slc25a46*** *(Wan et al., 2016)*	***Opa1*** *(Shahrestani et al., 2009)*, ***Dnm1l*** *(Altanbyek et al., 2016)*, ***Ssbp1*** *(Maier et al., 2001)*, ***Afg3l2*** *(Pareek and Pallanck, 2020)*, ***Slc25a46*** *(Ali et al., 2020)*	***Opa1*** *(Kanazawa et al., 2008)*, ***Ssbp1*** *(Sugimoto et al., 2008)*, ***Afg3l2*** *(Benedetti et al., 2006)*, ***Rtn4ip1*** *(Fujii et al., 2011)*, ***Dnm1l*** *(Scholtes et al., 2018)*
**Reduced visual acuity**	***Opa1*** *(Zaninello et al., 2020)*, ***Opa3*** *(Davies et al., 2008)*, ***Wfs1*** *(Bonnet-Wersinger et al., 2014)*	***Rtn4ip1*** *(Angebault et al., 2015)*, ***Wfs1*** *(Cairns, 2019)*	N/A	N/A
**Neuro-degeneration**	***Opa1*** *(Sarzi et al., 2012)*, ***Opa3*** *(Davies et al., 2008)*, ***Afg3l2*** *(Maltecca et al., 2009)*, ***Wfs1*** *(Waszczykowska et al., 2020)*	***Opa3*** *(Pei et al., 2010)*, ***Wfs1*** *(Cairns, 2019)*, ***Slc25a46*** *(Abrams et al., 2015)*	***Opa1*** *(Shahrestani et al., 2009)*, ***Wfs1*** *(Sakakibara et al., 2018)*, ***Afg3l2*** *(Pareek and Pallanck, 2020)*	N/A
**Motor defects**	***Opa1*** *(Sarzi et al., 2012)*, ***Opa3*** *(Davies et al., 2008)*, ***Afg3l2*** *(Maltecca et al., 2008)*, ***Mcat*** *(Smith et al., 2012)*	***Opa1*** *(Rahn et al., 2013)*, ***Opa3*** *(Pei et al., 2010)*, ***Slc25a46*** *(Abrams et al., 2015)*	***Opa1*** *(Shahrestani et al., 2009)*, ***Wfs1*** *(Sakakibara et al., 2018)*, ***Afg3l2*** *(Pareek and Pallanck, 2020)*, ***Slc25a46*** *(Suda et al., 2018)*	***Opa1*** *(Byrne et al., 2019)*, ***Dnm1l*** *(Scholtes et al., 2018)*
**Reduced size/BMI**	***Opa3*** *(Davies et al., 2008)*, ***Afg3l2*** *(Maltecca et al., 2008)*, ***Mcat*** *(Smith et al., 2012)*, ***Wfs1*** *(Kõks et al., 2009)*	***Wfs1*** *(Cairns, 2019)*	N/A	***Opa1*** *(Kanazawa et al., 2008)*, ***Rtn4ip1*** *(Knowlton et al., 2017)*
**Cardiac Defects**	***Ssbp1*** *(Jiang et al., 2021)*, ***Dnm1l*** *(Wakabayashi et al., 2009)*, ***Opa3*** *(Davies et al., 2008)*	***Opa1*** *(Rahn et al., 2013)*, ***Slc25a46*** *(Buglo et al., 2020)*, ***Ssbp1*** *(Del Dotto et al., 2020)*	***Opa1*** *(Shahrestani et al., 2009)*	N/A

*Created using Biorender.com.*

### Zebrafish-*Danio rerio* Models

*Danio rerio* (subsequently referred to simply as zebrafish), and particularly zebrafish larvae, are an emerging model in vision research. The stratification and function of the zebrafish retina is very similar to that of humans, with a comparable cone density. Zebrafish have four different cone types in comparison to the three cone types found in humans, conferring superior color vision, which is often affected in autosomal OA patients. Zebrafish possess RGCs which converge to form the optic nerve (also known as cranial nerve II in zebrafish), cross at the optic chiasm, and innervate the optic tectum, the visual center of the brain (Diekmann et al., 2015). Notably, RGCs in zebrafish exhibit intraretinal myelination, in contrast to rodents and primates in which myelination of the optic nerve begins a short distance outside of the globe of the eye. Unlike mammals, zebrafish can regenerate their optic nerve following chemical or mechanical damage, although this ability reduces somewhat with age (Münzel et al., 2014). It is unclear whether this will impede the pathogenic effects of autosomal OA-associated mutations. Zebrafish visual function can be readily assessed in larvae, principally through measuring the optokinetic (OKR) and visual motor responses (VMR), on a much larger scale than rodents (Emran et al., 2008; Zou et al., 2010). Zebrafish models are available for study much faster than rodent models with RGCs differentiating at 28 h post fertilization and OKR and VMR assays possible from 3 days post fertilization. At least 71% of human genes have an ortholog in zebrafish (Howe et al., 2013), with most genes demonstrating between 50 and 80% homology with their human equivalent (Broughton et al., 2001). Notably, there was a whole genome duplication event in the evolution of teleost fish (Pasquier et al., 2016) and consequently, many zebrafish homologs for mammalian genes are present in duplicate and may exhibit some degree of functional redundancy. This is the case for *Wfs1* (Cairns, 2019), and may confound results when recreating pathogenic mutations (Kleinjan et al., 2008; Lu et al., 2012). Zebrafish develop externally, making them accessible to genetic manipulation through microinjection. There are 2 principal methods of genetic manipulation in zebrafish embryos, CRISPR/Cas9 and morpholinos. Morpholinos are short oligomers designed to be complementary to the transcription start site or splice site of pre-mRNAs to transiently block splicing or translation of a desired transcript, reducing the expression of the target gene. Although the typical duration of the knockdown (usually a few days post-injection) would be sufficient for studies in larvae, it would not be sustained in adult fish (Rahn et al., 2013). Whilst this could be used to look at the developmental abnormalities implicated in some of the autosomal OA-associated genes, it may be unsuitable for looking at progressive, degenerative phenotypes. It should also be noted that as much as 20% of morpholinos exhibit non-specific toxicity, particularly relating to apoptotic cell death (Ekker and Larson, 2001; Robu et al., 2007). CRISPR/Cas9 editing technology has been extensively exploited in zebrafish, with some protocols reporting editing efficiencies over 90% (Hoshijima et al., 2019). This technique can be used to create stable, heritable Kos of genes, but can additionally be utilized to insert human transgenes, or replicate patient mutations. Finally, zebrafish have many well established transgenic reporter lines, a particularly effective approach in translucent zebrafish embryos. There are several examples of lines expressing fluorescent proteins under the control of retinal ganglion cell specific promoters, for example Atonal BHLH Transcription Factor 7 (*atoh7*; Masai et al., 2003). Multiple transgenic lines specifically targeting mitochondria are also available, notably zebrafish with mitochondrial expression of photo-activatable fluorescent protein (mito-PAGFP) which, after photoactivation, allows for the tracking of individual mitochondria within sensory neurons (Wehnekamp et al., 2019; Mieskes et al., 2020), and *MitoFish*, which has been used for intravital imaging of mitochondria in immobilized larvae (Paquet et al., 2014). It would be valuable to apply available transgenic approaches to the study of retinal ganglion cell mitochondrial structure and function in zebrafish disease models of autosomal-OA.

To date, most of the published zebrafish autosomal OA models are based on morpholino-based knockdown of gene expression, many of which present with abnormalities in eye and optic nerve morphology (Pei et al., 2010; Del Dotto et al., 2020). Although vision has only been addressed in a limited number of studies, reduced visual acuity has been noted (Angebault et al., 2015; Cairns, 2019). Common secondary symptoms of autosomal OA such as motor deficits (Rahn et al., 2013; Angebault et al., 2015) and inner ear abnormalities (Cairns, 2019; Del Dotto et al., 2020) have also been observed. Models frequently exhibit mitochondrial abnormalities including disrupted mitochondrial networks, respiratory deficiency, and reduced mitochondrial trafficking (Abrams et al., 2015; Eijkenboom et al., 2019). As in the murine models, many zebrafish homozygous for autosomal OA-causing genes exhibit developmental defects of the heart and brain (Wan et al., 2016; Buglo et al., 2020; Del Dotto et al., 2020), perhaps emphasizing the need for further research into any developmental origins for autosomal OA pathogenicity. Given the relative ease of generating novel zebrafish models, there is ample opportunity for creating models for autosomal OA-causing genes, in addition to examining degenerative phenotypes in adult fish. The extant zebrafish models could be well suited to drug screening, due to the prevalence of strong visual phenotypes from the early larval stage and relative ease with which visual acuity can be determined.

### *Drosophila* Models

The fruit fly, *Drosophila melanogaster*, is a well-established *in vivo* model system with at least 75% of disease-linked genes having *Drosophila* orthologs (Reiter et al., 2001). They are relatively inexpensive to rear, produce large numbers of offspring and have a maximal lifespan of approximately 3 months (Huang et al., 2020), which may prove useful in studying degenerative diseases. Of relevance to autosomal OA, flies have eyes, relatively complex brains and some organs which have comparable function to their human equivalents. A notable disadvantage of *Drosophila* as a model for autosomal OA is the significant disparity in eye morphology. Flies have compound eyes, comprising over 700 repeating units called ommatidia. Each ommatidium is composed of eight photoreceptors which do not converge to form a structure resembling the optic nerve (Paulk et al., 2013). Despite the lack of RGCs, *Drosophila* offer a strong platform for examining neurons *in vivo*. Their relatively simple organization allows for easily reproducible examination of the morphology and function of long axons *in situ* (Dolph et al., 2011). As with other models, CRISPR techniques have meant that targeted gene editing is now relatively straightforward in flies, though most published studies to date have employed knockdown of gene expression.

Efficient ubiquitous knockdown of most OA-associated genes is lethal, corroborating findings in the other animal models suggesting a developmental role for many autosomal OA genes. As such the UAS-GAL4 system, which allows for generation of tissue-specific expression of RNAis (Dolan et al., 2017; Ogienko et al., 2020), is commonly used to study the role of autosomal OA-causing genes by targeted knockdown in neurons. Locomotor issues are common, in some instances age-dependent and progressive, generally in keeping with mobility issues present in corresponding patients (Sakakibara et al., 2018; Suda et al., 2018; Pareek and Pallanck, 2020). The majority of fly autosomal OA-models do not have a gross eye phenotype, but the limited number of studies that investigated visual function have reported developmental and electrical response defects (Maier et al., 2001; Verstreken et al., 2005). Most published *Drosophila* models of autosomal OA-associated genes have highly abnormal mitochondria including: abnormal cristae, fragmented or aggregated mitochondrial networks, perturbed axonal trafficking, mtDNA depletion, hyperfused or elongated mitochondria, reduced ATP content, increased ROS, and reduced abundance and/or activity of complexes of the electron transport chain, generally replicating the perturbation seen in patients (Tang et al., 2009; Pareek and Pallanck, 2020). Similar to the mice, some fly models exhibit heart tube abnormalities, despite abnormal cardiac development not being prevalent in patients (Shahrestani et al., 2009; Bhandari et al., 2015). Overall, the few existing *Drosophila* autosomal OA models present a strong platform for examining mitochondrial and neurodegenerative phenotypes associated with autosomal OA genes.

### *Caenorhabditis elegans* Models

Like *Drosophila*, the roundworm *Caenorhabditis elegans* has been an invaluable asset to biological research generally, as an extremely simple and abundant organism. *C. elegans* is inexpensive to rear, can produce up to 1,000 offspring, has an extremely short lifespan of ∼20 days, and may be kept in frozen storage over long periods of time and revived (Vita-More and Barranco, 2015). Human homologs exist for at least 80% of the *C. elegans* proteome (Lai et al., 2000) with known worm homologs for most autosomal OA genes, with the exception of *WFS1*. *C. elegans* does not possess eyes, retinas, or opsin pigments, and therefore has no structure that directly parallels the optic nerve or retinal ganglion cells. Instead, it perceives UV light using its distinct LITE-1 photoreceptor (Edwards et al., 2008; Gong et al., 2016), Due to this disparity between the human and nematode visual systems, they have limited use in directly modeling visual defects associated with autosomal OA. Nevertheless, *C. elegans* presents an interesting model for the study of mitochondrial morphology and dynamics, particularly within neurons, due to their simplistic and transparent body plan facilitating a wide array of microscopy techniques. Furthermore, as gene editing in *C. elegans* can be as straightforward as feeding sgRNA to transgenic animals (Liu et al., 2014), combined with the comparatively small expense of roundworms as a model organism, this could be a highly accessible avenue for expanding the field of autosomal OA research.

Many *C. elegans* mutants for autosomal OA-linked genes exist (Table 2), however many were created during large mutagenesis or RNAi screens. Despite this, there are several phenotypes seen within the *C. elegans* autosomal OA models that are conserved within higher eukaryotic organisms, notably abnormal mitochondrial morphology often accompanied by irregular cristae (Knowlton et al., 2017; Machiela et al., 2020), respiratory defects (Fujii et al., 2011; Luz et al., 2015), embryonic or larval lethality (Zubovych et al., 2010; Nargund et al., 2012), locomotor dysfunction (Machiela et al., 2020), and neuronal dysfunction (Zaninello et al., 2020). There are some notable disparities between the vertebrate and *C. elegans* autosomal OA models. While aberrant expression of autosomal OA genes frequently reduces maximal lifespan in *Drosophila* or vertebrate models, in some worm autosomal OA models lifespan is extended (Kanazawa et al., 2008; Chaudhari and Kipreos, 2017; Maglioni et al., 2019). This may be due to the ability of C. elegans to arrest development in the dauer state in response to adverse environmental conditions. Dauer larvae are highly resistant to all forms of stress, including oxidative stress, favor anaerobic respiration, and are comparatively long-lived. In at least two studies arrest in the dauer state was observed (Avery, 1993; Maglioni et al., 2019). As with all studies involving model systems, careful consideration must be given to ensure that the system used is appropriate for the research question being investigated.

### Retinal Organoids

Much of our understanding of human biology has stemmed from research involving animal model organisms like those discussed previously. They have played a substantial role in informing many important scientific discoveries over the years, however, there are many fundamental differences that exist between humans and their model organism counterparts (Huch et al., 2017), especially in the context of optic neuropathies. Recent studies have highlighted significant differences in both the number and distribution of RGCs in rodents compared to primates (Peng et al., 2019) and this may explain why therapeutic approaches that appear to show great promise in animal models do not always translate effectively to human patients, highlighting the need for an accurate *ex vivo* model. The discovery of induced pluripotent stem cells (iPSCs) has enabled biologists to reprogram human somatic cells to a pluripotent, embryonic stem cell-like state (Takahashi et al., 2007), which, combined with recent improvements in three dimensional culture techniques has led to the emergence of organoid models as a potential avenue to remedy this issue.

An “organoid” can be simply defined as a structure that resembles an organ (Dutta et al., 2017). Derived from either pluripotent stem cells or organ progenitor cells, they undergo differentiation, forming organ-like structures in a process which appears to mimic *in vivo* organogenesis (Dutta et al., 2017). The first protocol for the directed differentiation of retinal organoids was published in 2012 (Nakano et al., 2012) and since then a variety of approaches have been developed (Chen et al., 2016; Chichagova et al., 2019). The differentiation process results in the formation of retinal organoids that contain all of the major retinal cell types, organized in a laminar manner which emerge at roughly the same time points as in the human retina (Capowski et al., 2019; Chichagova et al., 2019). RGCs are the first distinct cell population to emerge during retinal development and this is mirrored in organoid development (Rabesandratana et al., 2018). Differential expression of *OPN4* reveals the presence of melanopsin expressing-photosensitive RGCs at as early as 5 weeks of differentiation, with positive immunostaining for the RGC-specific markers Smi32 and RBPMS indicating their orientation in the basal aspect of the organoid (Mellough et al., 2019), reminiscent of the native retina. Mitochondrial function can also be studied with relative ease in retinal organoids through the use of transmission electron microscopy (Duong et al., 2021) and the use of bioassays measuring viability (Das et al., 2020), ATP production (Duong et al., 2021) and oxygen consumption rate (Roy-Choudhury and Daadi, 2019). These organoids are capable of forming functional synapses and are responsive to light stimulation (Mellough et al., 2015), however, they still lack many features of the native retina – such as vasculature (Laschke and Menger, 2012) and the presence of microglial cells (Fathi et al., 2021) – which can somewhat limit their use in the context of modeling optic neuropathies. Retinal organoids are becoming an increasingly attractive model for the study of inherited ocular disorders as they provide researchers with the unique opportunity to model patient-specific mutations through the use of iPSCs (Bell et al., 2020). As with *in vivo* models, CRISPR/Cas9 gene-editing technology can also be applied, either to introduce *de novo* mutations or to generate isogenic controls by correcting mutations in patient-derived iPSC lines (VanderWall et al., 2020). While disease modeling through the use of retinal organoids is highly applicable, there are currently no published examples of retinal organoids being used to model OA.

The main advantage of disease modeling through the use of retinal organoids is that they provide researchers with the opportunity to study human disease within a human model system while simultaneously circumventing the ethical challenges surrounding the study of early embryonic development in humans. Organoids are highly amenable to drug screening and to genetic manipulation, meaning they have a wide range of potential applications within this field. However, there are still many issues that need to be addressed in order for retinal organoids to reach their full potential. Despite being able to produce all of the major retinal cell types (Chichagova et al., 2019), as time in culture is extended the innermost layers of the organoid containing the RGCs begin to degenerate (Fathi et al., 2021). This is likely due to the fact that there is no vasculature present in the organoid, so as it grows bigger the amount of oxygen and nutrients that can diffuse through to the inner layers is not sufficient to maintain viability (Fathi et al., 2021), while spatial constraints imposed by the culture vessels in which organoids are grown mean that RGC axons cannot extend to the length they normally would in the native retina (Ohlemacher et al., 2019). In addition to this, microglial cells, which would ordinarily interact with Müller glial cells to regulate neuronal development (Li et al., 2019) are absent (Fathi et al., 2021), while mature structures such as an optic nerve are also not present. Retinal organoids have already proven to be a valuable tool for studying development and disease, and hold huge therapeutic potential from the perspective of regenerative medicine (Kobayashi et al., 2018; Eastlake et al., 2019). They can overcome limitations of *in vivo* disease models in that being a human model system removes the need to account for cross-species differences and could also bypass the issue of homozygous lethality caused by the introduction of OA-causing mutations seen in many of the other models. The ability to produce fully functional, mature retinal organoids in combination with gene-editing technologies such as CRISPR could hold the key to the treatment of inherited neuropathies such as autosomal OA. Research in this field is constantly evolving with the development of co-culture methods to promote RGC axon growth (Fligor et al., 2021) and the potential use of CRISPR reporter iPSC lines making it possible to produce organoids that resemble their human organoid counterparts even more closely.

The conservation of mitochondrial aberrations and phenotypic symptoms seen in both animal models and patients supports the causative relationship between OA pathology and mitochondrial dysfunction. Additionally, as visual pathology is often able to be replicated, specifically in zebrafish and mice, through expression of OA-causing gene mutations, this indicates that RGC vulnerability likely relates to common anatomical and molecular features within these models. This is especially relevant as research is underdeveloped in this area. There is some disparity between the animal models discussed here and patients, for example heart and brain developmental abnormalities (Angebault et al., 2015). Retinal organoids derived from human cells could be a useful addition to the field for the study of human RGCs, however it is clear that there is significant refinement required before they are sufficiently able to replicate *in vivo* human RGC morphology and function. The model organisms discussed here already have assays and histological techniques which would be readily applicable to the study of autosomal OA: *Drosophila* and *C. elegans* are well suited to provisionally studying perturbations in mitochondrial and neuronal function, whilst mice and zebrafish are highly applicable for examining changes in RGCs and visual acuity. Lastly, there is currently a paucity of studies investigating genetic or therapeutic modifiers of OA-associated degeneration, despite the abundance of methods to do so in all species mentioned. This is of eminent importance for future development in this field due to the lack of treatment options available for patients.

## Concluding Remarks

Autosomal OA is a highly genetically heterogeneous condition, with a growing list of genes which have been shown to give rise to the disorder. Despite this, as we have made clear in this review, there is an evident commonality with all presently implicated genes involved in the function and organization of mitochondria. It is unclear whether a particular aspect of mitochondrial function makes RGCs vulnerable, or induces cell degeneration, however this may be further elucidated through examining common pathways between affected genes, and common dysfunction caused by pathogenic mutations. Although various animal models bearing mutations for autosomal OA-genes exist, many recreate mutations associated with other diseases. Given the accessibility of CRISPR/Cas9 gene editing technologies, this could be easily remedied. Finally, retinal organoids present an exciting intermediary between *in vitro* and *in vivo* model systems, which could be used for the study of autosomal OA-associated pathological changes within the human eye. Any of the models discussed here could be utilized for drug screening so that we might begin to develop much needed treatment strategies for retinal disease.

## Author Contributions

ES, DM, AR, BK, and NO’S wrote and edited the manuscript. ES prepared the figures. JC commented on and reviewed the manuscript prior to submission. All authors contributed to manuscript revision, read and approved the submitted version.

## Conflict of Interest

The authors declare that the research was conducted in the absence of any commercial or financial relationships that could be construed as a potential conflict of interest.

## Publisher’s Note

All claims expressed in this article are solely those of the authors and do not necessarily represent those of their affiliated organizations, or those of the publisher, the editors and the reviewers. Any product that may be evaluated in this article, or claim that may be made by its manufacturer, is not guaranteed or endorsed by the publisher.
